# The complete chloroplast genome of *Ophiopogon japonicus*, an ornamental and medicinal plant

**DOI:** 10.1080/23802359.2019.1659110

**Published:** 2019-09-03

**Authors:** Can Yuan, Fang Peng, Shan Tao, Xiu-Fen Sha, Miao Xiong, Yuan-Yuan Chen, Fang-Sheng Mu, Chao Zhang

**Affiliations:** Industrial Crop Research Institute, Sichuan Academy of Agricultural Sciences, Chengdu, China

**Keywords:** *Ophiopogon japonicus*, chloroplast genome, phylogenetic analysis

## Abstract

*Ophiopogon japonicus*, extensively distributed in East Asia, is widely used in landscaping, the tuber of which also play a vital role in Oriental Medicine. Here, we reported the complete chloroplast genome which exhibited a typical quadripartite structure, 156,679 bp in length with 37.7% overall GC content, including 131 protein-coding genes, 37 transfer RNA genes, eight ribosomal RNA genes, and one pseudogene. Phylogenetic analysis suggested that *O. japonicus* has a close relationship to *Liriope spicata*.

*Ophiopogon japonicus*, also known as Mondo grass, an evergreen perennial grass that belongs to the *Liliaceae* family of which the phylogeny is a long-standing and challenging problem (Patterson and Givnish 2002), has been wildly used as ground cover due to its high tolerance to shade and barren (Fantz 2009). The dry tuber of *O. japonicus*, a famous traditional Chinese medicine material (called as Maidong in Chinese) has great medicinal efficacy in the therapy of inflammatory, cough, cardiovascular, etc., caused by yin deficiency due to the active constituent contained, such as polysaccharide, homoisoflavonoids, and so on (Kou et al. 2005). The production from Mianyang area, Sichuan province, China, was traditionally recognized as the authentic and superior herb. However, up to now, the available sequences for taxonomic status investigation of *O. japonicus* are critically scarce which not only hamper the gene pool utilization but also impose restrictions on phylogeny inferring for *Liliaceae* family and *Ophiopogon*. Thus, we sequenced the chloroplast sequence of *O. japonicus* to shed the light on genetic improvement for *O. japonicus* and relationships resolving for *Liliaceae* family.

Using a modified CTAB method (Doyle 1987), DNA was extracted from the young and fresh leaf of Chuanmaidong 1 (one excellent variety of *O. japonicus* planted in scientific research base of Industrial Crop Research Institute, Sichuan Academy of Agricultural Sciences (Qingbaijiang District, Chengdu, China), for long-term conservation). The specimen was collected from Santai county, Sichuan province (E31.2734611750, N104.9409004795). The specimen (No. Chuanmaidong 1) is stored in the Crop Germplasm Bank of Industrial Crop Research Institute, Sichuan Academy of Agricultural Sciences. Employing the long-range PCR strategy and the quality and concentration met DNA as a template, the whole chloroplast genome was amplified by nine universal primer pairs (Yang et al. 2014). Then, PCR production was subjected to library construction and sequenced by the high-throughput platform of Illumina HiSeq 2500. The chloroplast genome was assembled using SOAP denovo2 (Luo et al. 2015) based on a close species sequence as reference. Subsequently, annotations were executed via online software DOGMA (Wyman et al. 2004) and manually corrected. The sequence and annotation information were submitted to NCBI (MK952744).

With 156679 bp in length and 37.7% overall GC content, the complete chloroplast genome possesses a typical circularly quadripartite structure, which is commonly characterized among angiosperm, a large single-copy (LSC) region of 84,939 bp and a small single-copy (LSC) region of 18,036 bp which are separated by two inverse repeat region 26,852 bp and the GC content of each part is 35.7%, 31.8%, and 42.8%, respectively. The cpDNA of *O. japonicus* comprised 177 genes, including 131 protein-coding genes, 37 transfer RNA genes, eight ribosomal RNA genes, and one pseudogene.

Further, combining with available chloroplast genome of close species within *Liliaceae* family, a phylogenetic tree was constructed based on the maximum likelihood method using RAxML (Stamatakis 2014). The result validated a close relationship of *Liriope spicata* and *O. japonicus* as a sister clade following *Convallaria keiskei* received a strong support value and presented an explicit phylogeny of *Liliaceae* family (Figure 1).

**Figure 1.**
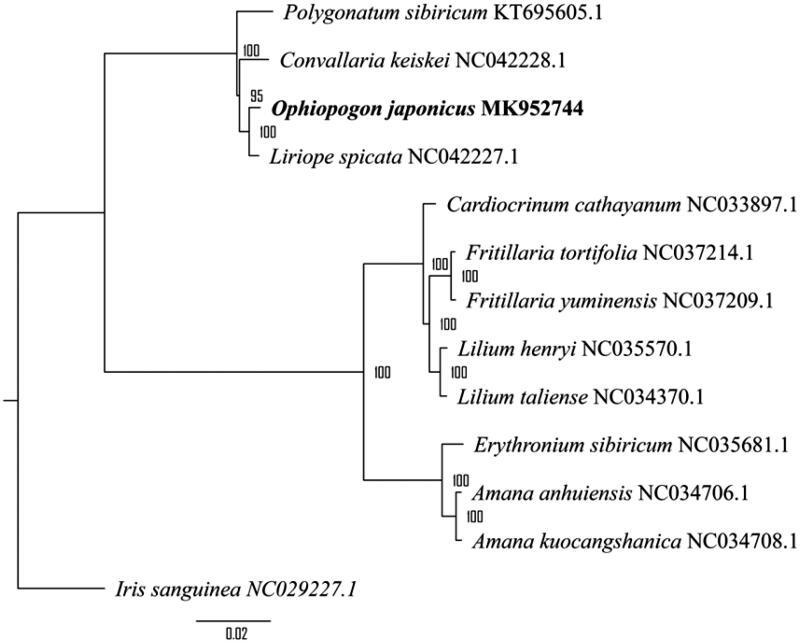
Maximum-likelihood (ML) phylogenetic tree: the number showed on node stand for bootstrap value on 1000 replicates; *Iris sanguinea* is set as outgroup.
